# Role of the Gut Microbiome in Natural and Synthetic Drug Efficacy and Safety: A Narrative Review

**DOI:** 10.3390/applmicrobiol6100113

**Published:** 2026-09-24

**Authors:** Ricardo Villoch-Rosado, Dijanneliz Acosta, Lorelein Morales-Santiago, Isabel M. González-Tardí, Omaira Medina-Rodríguez, Karoline Ríos-Rodríguez, Ivette Agront-Medina, Alejandra E. Alvarado-Andino, Luis A. Báez-Rodríguez, Alberto Calderón-Nuñez, Adneliz G. Cátala-Cátala, Nomarys Delgado-Negrón, Tayra L. Figueroa-Reyes, Luis Mojica-Pérez, Héctor Rosa-López, Andrés Santos-Bermúdez, José Rodríguez-Zayas, Eduardo L. Tosado-Rodríguez

**Affiliations:** 1 Toxicology and Drug Design Program, Department of Science and Technology, Universidad Ana G. Méndez (UAGM), Cupey, P.O. Box 21150, San Juan, PR 00928-1150, USA; 2 School of Dental Medicine, Universidad Ana G. Méndez (UAGM), Gurabo Campus, Gurabo, P.O. Box 21150, San Juan, PR 00928-1150, USA

**Keywords:** gut microbiome, gut microbiota, pharmacomicrobiomics, drug metabolism, microbiome–drug interactions, cancer, neurological disorders, psychiatric disorders, metabolic diseases, natural products

## Abstract

The gut microbiome has emerged as an important determinant of the metabolism, efficacy, and safety of many therapeutic agents, expanding the traditional host-centered view of pharmacology. Direct microbial biotransformation is particularly relevant to orally administered compounds, whereas the gut microbiome can also influence systemically administered therapies indirectly through immune, inflammatory, barrier, and hepatic pathways. Beyond its established roles in nutrient metabolism, immune regulation, and maintenance of epithelial homeostasis, the microbiome functions as a metabolically active organ capable of transforming endogenous molecules, dietary compounds, xenobiotics, and therapeutic agents through an extensive repertoire of microbial enzymes and metabolites. These interactions influence virtually every stage of drug disposition by modifying pharmacokinetics, pharmacodynamics, bioavailability, therapeutic efficacy, and toxicity. This narrative review summarizes current knowledge of the composition and functional diversity of the gut microbiome and examines the molecular mechanisms underlying host–microbe–drug interactions, including direct microbial biotransformation, regulation of intestinal barrier integrity, immune modulation, bile acid metabolism, and microbial control of hepatic drug-metabolizing pathways. We further discuss the impact of these mechanisms across multiple therapeutic areas, including oncology, neurological and psychiatric disorders, cardiovascular medicine, diabetes, metabolic diseases, and microbiome-dependent activation of natural products. Emphasis is placed on recent advances in cancer therapy, where the microbiome has emerged as a key determinant of responses to chemotherapy, immune checkpoint inhibitors, oncolytic viruses, and other immunotherapies. The review also highlights the bidirectional relationship between drugs and microbial communities, illustrating how pharmaceuticals reshape the microbiome while microbial ecosystems simultaneously influence therapeutic outcomes. Finally, we discuss emerging technologies, including multi-omics approaches, artificial intelligence, organ-on-chip models, and microbiome-targeted interventions, that are accelerating the integration of pharmacomicrobiomics into drug discovery and precision medicine. Collectively, current evidence supports recognizing the microbiome as an integral component of human pharmacology and a promising source of predictive biomarkers and therapeutic targets for the development of safer, more effective, and personalized treatment strategies.

## 1. Introduction

The human microbiome is a highly dynamic ecosystem composed of bacteria, archaea, fungi, viruses, and protozoa that colonize virtually every epithelial surface of the human body. In this review, we follow current terminological convention by using “microbiota” to refer specifically to the community of living microorganisms themselves—their taxonomic composition, abundance, and diversity—and “microbiome” to refer to the collective genomes, functional gene content, and metabolic capacity of that community together with its local environment; the two terms are therefore not used interchangeably throughout the manuscript. Rather than functioning as passive commensals, these microbial communities actively regulate nutrient metabolism, immune maturation, epithelial barrier integrity, and host signaling pathways, making them integral to human physiology and disease susceptibility [1,2]. Distinct microbial niches, including the gastrointestinal tract, oral cavity, skin, respiratory tract, and urogenital tract, harbor unique taxonomic and functional profiles shaped by age, diet, environmental exposures, medication use, and host genetics [3]. Advances over the past decade have also challenged the traditional view of microbes as by-standers in chronic disease. Instead, they are increasingly recognized as active participants in metabolic disorders, immune dysregulation, neurological diseases, and cancer, where they influence disease initiation, progression, and therapeutic response [4]. This growing body of evidence has fundamentally shifted microbiome research from descriptive ecology toward mechanistic investigations that seek to understand how microbial functions shape clinical outcomes.

This shift has been driven largely by technological advances. Culture-based approaches and clone library sequencing provided the first insights into microbial diversity but captured only a small fraction of the microorganisms inhabiting the human body. The integration of shotgun metagenomics, metatranscriptomics, metaproteomics, metabolomics, culturomics, and increasingly sophisticated computational frameworks has transformed our ability to characterize microbial communities and, more importantly, their functional capacity [5]. These multi-omics strategies have revealed that the microbiome possesses a collective metabolic repertoire far exceeding that of the human genome, enabling microorganisms to synthesize bioactive metabolites, regulate host signaling pathways, and directly metabolize xenobiotics before they reach systemic circulation [6,7]. As a result, the microbiome is increasingly regarded as a metabolically active organ whose contribution must be considered when studying human physiology and pharmacology.

Among the different microbial habitats, the gut microbiome has received the greatest attention because of its enormous metabolic capacity and its intimate interaction with dietary compounds, pharmaceuticals, and the immune system. The intestine represents the first major site where many orally administered natural and synthetic compounds encounter an extensive microbial enzymatic network capable of activating prodrugs, inactivating therapeutics, generating toxic metabolites, or altering compound bioavailability before intestinal absorption [2,3]. At the same time, pharmaceuticals can reshape microbial composition and function, creating a bidirectional relationship in which drugs modify the microbiome while the microbiome determines therapeutic response, a concept now widely recognized as pharmacomicrobiomics [5,6]. This interaction extends beyond classical pharmacokinetics. Microbial metabolites regulate host immune responses, intestinal permeability, inflammatory signaling, and hepatic drug-metabolizing enzymes, collectively influencing efficacy, toxicity, and inter-individual variability across numerous therapeutic classes, including anticancer agents, immunotherapies, cardiovascular drugs, antidepressants, and antidiabetic medications [7,8].

The clinical implications of these interactions have become particularly evident in oncology, where differences in microbiota composition have been associated with variability in chemotherapy efficacy, immune checkpoint inhibitor response, treatment-related toxicity, and the emergence of drug resistance [9–11]. Similar observations are now emerging across multiple therapeutic areas, supporting the idea that microbial composition and function may become valuable biomarkers for predicting treatment response and identifying patients at greater risk of adverse drug reactions. Equally important, these discoveries have stimulated interest in microbiome-directed interventions, including probiotics, prebiotics, dietary modification, fecal microbiota transplantation, microbial enzyme inhibitors, and engineered microbial therapeutics, as complementary strategies to improve drug performance and reduce toxicity [8,12].

The rapid expansion of pharmacomicrobiomics research over the past decade reflects growing recognition of the microbiome as a major determinant of therapeutic response (Figure S1). Foundational microbiome studies have established that this complex microbial ecosystem is essential for digestion, host metabolism, immune regulation, and disease susceptibility [13,14]. More recently, pharmacomicrobiomic research has demonstrated that microbial communities also influence drug bioavailability, pharmacokinetics, pharmacodynamics, therapeutic efficacy, and toxicity, positioning the microbiome as a critical determinant of therapeutic outcomes, particularly for orally administered drugs and treatments that rely on immune or metabolic pathways [3,5,15,16]. In this narrative review, we examine the growing evidence that the microbiome shapes drug metabolism, efficacy, and safety across both natural compounds and synthetic therapeutics. We discuss the composition and functional capabilities of the human microbiome, the molecular mechanisms governing host–microbe–drug interactions, and the translational implications of these interactions for precision medicine, pharmaceutical research, and drug development. Finally, we highlight current challenges, emerging technologies, and future directions for integrating microbiome-informed strategies into next-generation therapeutics.

Although previous reviews have examined individual components of pharmacomicrobiomics, including microbial drug metabolism, cancer immunotherapy, the gut–brain axis, or specific drug classes, the literature remains fragmented across therapeutic disciplines. The distinctive objective of this narrative review is therefore to integrate direct microbial biotransformation with indirect host-mediated mechanisms and bidirectional drug–microbiome interactions within a unified framework encompassing both synthetic pharmaceuticals and natural products. Rather than cataloging microbiome–drug associations alone, we critically examine the level of evidence supporting these interactions across oncology, neurological and psychiatric disorders, cardiovascular disease, metabolic disease, and natural products, while highlighting interindividual variability, methodological limitations, translational barriers, and emerging microbiome-targeted strategies. This integrated perspective is intended to identify both well-supported mechanisms and areas where current evidence remains associative, preclinical, or insufficient for clinical implementation.

### Literature Search and Analysis Strategy

A narrative literature search was conducted using PubMed/MEDLINE, Scopus, and Web of Science from database inception through July 2026. The primary search strategy combined terms related to the gut microbiome and pharmacological outcomes, including “gut microbiome,” “gut microbiota,” “intestinal microbiome,” and “intestinal microbiota” with “drug metabolism,” “pharmacomicrobiomics,” “pharmacokinetics,” “pharmacodynamics,” “drug efficacy,” “drug toxicity,” and “therapeutic response.” Targeted searches were additionally performed for major therapeutic areas addressed in this review, including cancer, immunotherapy, chemotherapy, cardiovascular disease, diabetes and metformin, neurological and psychiatric disorders, and natural products or phytochemicals. Peer-reviewed English-language human studies, clinical trials, observational studies, and relevant animal or mechanistic investigations directly addressing gut microbiome–drug or gut microbiome–natural product interactions were considered for inclusion. High-quality reviews and meta-analyses were also used to provide contextual background and to identify additional relevant primary studies. Studies unrelated to therapeutic response, microbiome studies without direct pharmacological relevance, duplicate publications, conference abstracts lacking sufficient methodological or outcome information, and studies focused exclusively on non-gut microbial communities were generally excluded unless they provided necessary contextual comparison. Because this was a narrative rather than systematic review, no formal meta-analysis or standardized risk-of-bias instrument was applied. Instead, evidence was critically evaluated according to study design, biological plausibility, reproducibility across independent studies, human versus preclinical evidence, sample size when relevant, and consistency of reported microbiome–drug associations.

## 2. Composition and Functional Capacity of the Gut Microbiome

Health depends not simply on microbial diversity but on the preservation of functional equilibrium within these microbial communities. When this balance is disrupted, a condition commonly referred to as dysbiosis; critical microbial functions are altered, often resulting in chronic inflammation, impaired immune regulation, metabolic dysfunction, and increased disease susceptibility. Dysbiosis has been associated with numerous disorders, including inflammatory bowel disease, type 1 diabetes, multiple sclerosis, HIV infection, cardiovascular disease, and multiple cancer types [1,17]. Importantly, dysbiosis should not be viewed solely as the loss or gain of individual microbial species. Current evidence suggests that disruption of microbial metabolic pathways, ecological interactions, and metabolite production may be equally important for determinants of disease development and therapeutic response [18–20]. This functional perspective has become particularly relevant in pharmacomicrobiomics, where microbial enzymatic activity can substantially influence the metabolism, efficacy, and toxicity of natural products and synthetic drugs [5,6].

Beyond dysbiosis itself, gut microbiome-mediated drug response is highly individualized. Interindividual differences in microbial strain composition, microbial gene content, metabolic capacity, and baseline community structure can determine whether a particular drug-metabolizing pathway is present or functionally active. Host genetics further contributes to this variability by influencing immune function, intestinal physiology, bile acid metabolism, and the ecological niches that shape microbial colonization. Disease-related dysbiosis introduces an additional layer of complexity because the disease being treated may itself alter microbial composition and function, making it difficult to distinguish whether a microbial signature is a cause of altered therapeutic response, a consequence of disease, or both. Together with diet, age, antibiotic exposure, geography, environmental factors, lifestyle, and previous treatment history, these factors complicate the identification of universal microbial biomarkers and support functional, longitudinal, and patient-specific approaches to pharmacomicrobiomics.

Non-gastrointestinal niches, including the oral, respiratory, skin, and vaginal microbiomes, perform site-specific immune and barrier functions and have been linked to periodontal disease [21–23], chronic obstructive pulmonary disease and asthma [24,25], inflammatory skin disorders, and reproductive or gynecological conditions [26–28], respectively; more broadly, oral microbial communities also shape systemic health outcomes [29,30]. Because the pharmacomicrobiomic mechanisms reviewed here are predominantly gastrointestinal, these niches are not discussed further.

Among these microbial ecosystems, the gut microbiome exerts the broadest systemic influence owing to its extraordinary metabolic capacity and continuous interaction with dietary compounds, xenobiotics, and the host immune system. Beyond its established roles in digestion, vitamin synthesis, including vitamin K, and maintenance of intestinal barrier integrity, the gut microbiota produces a wide range of bioactive metabolites, including short-chain fatty acids, secondary bile acids, and amino acid derivatives, that regulate inflammatory pathways, immune cell differentiation, and host metabolism [31–34]. These microbial functions influence diseases extending well beyond the intestine, including colorectal cancer, cardiovascular disease, acute pancreatitis, metabolic disorders, and autoimmune conditions [1,35,36]. Equally important, the extensive enzymatic repertoire of the gut microbiota enables direct biotransformation of numerous endogenous and exogenous compounds, establishing a mechanistic link between microbial ecology and drug disposition [37,38]. Recognition of the microbiome as an active participant in xenobiotic metabolism has fundamentally expanded our understanding of pharmacology [39] and provides the biological foundation for the emerging field of pharmacomicrobiomics, in which individual microbial composition and function are increasingly recognized as determinants of therapeutic efficacy, toxicity, and personalized treatment response [5,7,8] (Figure 1).

## 3. Microbiome–Drug Interactions

### 3.1. The Microbiome as a Metabolic Organ

The human gastrointestinal microbiome is increasingly recognized as a metabolically active organ that extends the biochemical capabilities of its host. While the liver has traditionally been viewed as the principal site of xenobiotic metabolism, it is now evident that orally administered compounds encounter an extensive microbial enzymatic network before reaching systemic circulation. This microbial community, composed of trillions of microorganisms and an estimated 3.3 million nonredundant genes, encodes a metabolic repertoire that exceeds the human genome by nearly two orders of magnitude, enabling biochemical transformations that human tissues alone cannot perform [6,7]. Consequently, the intestine should no longer be regarded merely as a passive absorptive surface but as a dynamic metabolic interface where microbial and host enzymes collectively determine the fate of endogenous molecules, dietary constituents, environmental xenobiotics, and therapeutic agents (Figure 1).

This expanded metabolic capacity has fundamentally changed our understanding of pharmacology. Drug disposition has historically been interpreted through host-centered processes encompassing absorption, distribution, metabolism, and excretion (ADME) [40,41]. However, this framework does not account for the substantial contribution of intestinal microorganisms, which frequently metabolize orally administered compounds before hepatic first-pass metabolism occurs. Depending on the microbial species and enzymes involved, these transformations may activate inactive prodrugs, inactivate therapeutically active compounds, generate toxic metabolites, alter physicochemical properties such as hydrophobicity and solubility, or compete with host metabolic pathways for the same substrates [5–7]. The consequence is substantial interindividual variability in drug efficacy and toxicity that cannot be explained by pharmacogenomics alone (Figure 1).

Recognition of these interactions has given rise to pharmacomicrobiomics, a discipline that investigates how the composition and functional activity of the microbiome influence pharmacokinetics, pharmacodynamics, and therapeutic response [42–44]. Unlike pharmacogenomics, which focuses on inherited variation within the human genome [45], pharmacomicrobiomics incorporates the collective genetic potential of host-associated microorganisms as an additional source of biological variability. Importantly, microbial communities are highly dynamic. Diet, age, antibiotic exposure, disease, geography, environmental factors, and medication history continuously reshape microbial composition and metabolic activity, producing temporal fluctuations in drug response even within the same individual [5,35,46,47]. This dynamic nature makes the microbiome an attractive therapeutic target because, unlike the human genome, it can be intentionally modified.

Antibiotics represent the most obvious example of this bidirectional relationship, yet numerous non-antimicrobial medications, including proton pump inhibitors, metformin, statins, selective serotonin reuptake inhibitors, laxatives, antipsychotics, and chemotherapeutic agents, consistently alter microbial diversity, abundance, and metabolic function. These drug-induced ecological shifts may subsequently influence the metabolism of concurrently administered medications, creating feedback loops that contribute to treatment variability, cumulative toxicity, or therapeutic failure.

This emerging perspective has important implications for drug discovery and precision medicine. Rather than considering the microbiome solely as a confounding variable, it should be viewed as an integral component of human pharmacology that influences therapeutic response throughout the drug lifecycle, from preclinical development to clinical implementation. Advances in metagenomics, metabolomics, culturomics, transcriptomics, and systems pharmacology have made it possible to characterize microbial functions with unprecedented resolution, enabling researchers to identify microbial biomarkers associated with treatment response and to design microbiome-informed therapeutic strategies [7,8]. These technologies are driving a transition from descriptive associations toward mechanistic understanding, ultimately supporting the development of precision therapeutics that account for both host genetics and microbial metabolism (Figure 1).

### 3.2. Mechanisms of Microbiome–Drug Interactions

Drug–microbiome interactions occur through a complex network of direct microbial enzymatic reactions and indirect host-mediated mechanisms. Although these processes are often discussed separately, they operate simultaneously and frequently influence one another. Direct mechanisms involve microbial enzymes that chemically modify therapeutic compounds before absorption or during enterohepatic circulation, whereas indirect mechanisms arise from microbial regulation of host physiology, including immune responses, epithelial barrier integrity, bile acid metabolism, and hepatic drug-metabolizing enzymes. Together, these interactions influence virtually every stage of drug disposition, including absorption, distribution, metabolism, efficacy, toxicity, and elimination [5–7].

Among these mechanisms, direct microbial metabolism is currently the best characterized. The intestinal microbiota encodes thousands of enzymes capable of catalyzing reduction, hydrolysis, deconjugation, dehydroxylation, acetylation, demethylation, decarboxylation, and oxidation reactions that modify drug structure before systemic absorption [3,5,8,12,48,49]. These reactions are functionally analogous to hepatic phase I and phase II metabolism, although they are catalyzed by entirely different enzymatic systems [6,7,48,50]. Representative microbial enzymes include β-glucuronidases, azoreductases, nitroreductases, sulfatases, bile salt hydrolases, β-lyases, and tyrosine decarboxylases, each capable of altering drug activity through highly specific biochemical transformations [6–8,12,50].

These reactions can produce markedly different pharmacological outcomes. In some cases, microbial metabolism activates therapeutically beneficial compounds, as exemplified by sulfasalazine, whose azo bond is cleaved by bacterial azoreductases to release the active anti-inflammatory agent 5-aminosalicylic acid [51]. In contrast, microbial enzymes may inactivate drugs, reducing therapeutic efficacy. One of the best-characterized examples involves *Eggerthella lenta*, which expresses the cardiac glycoside reductase (cgr) operon capable of reducing digoxin into inactive dihydrodigoxin, thereby decreasing drug effectiveness in susceptible individuals [52]. Likewise, *Enterococcus faecalis* expresses tyrosine decarboxylase that converts levodopa into dopamine within the intestinal lumen, while *Eggerthella lenta* subsequently metabolizes dopamine into m-tyramine, substantially reducing levodopa bioavailability before it reaches the central nervous system [53–55]. These discoveries illustrate how individual microbial species, and even specific microbial genes, can determine treatment response independently of host genetics.

Equally important is microbial participation in enterohepatic recycling. Drugs conjugated in the liver through glucuronidation are often excreted into the intestine, where microbial β-glucuronidases hydrolyze glucuronide conjugates, regenerating the parent compound for reabsorption [56,57]. While this process prolongs the activity of certain medications, it may also increase toxicity. Irinotecan provides one of the most clinically relevant examples. Following hepatic glucuronidation of its active metabolite SN-38, microbial β-glucuronidases regenerate active SN-38 within the intestine, leading to epithelial injury and severe delayed diarrhea, a dose-limiting adverse effect that has stimulated the development of selective microbial β-glucuronidase inhibitors as adjunctive therapies [58,59]. Similar microbial transformations have been described for nonsteroidal anti-inflammatory drugs, estrogens, morphine, mycophenolate mofetil, and numerous dietary phytochemicals, illustrating that microbial metabolism is not an isolated phenomenon but a fundamental determinant of drug disposition [3,5,8,34,60–63].

### 3.3. Indirect Mechanisms of Microbiome–Drug Interactions

While direct microbial biotransformation has provided many of the landmark discoveries in pharmacomicrobiomics, it represents only one component of a much broader network of host–microbiome interactions. The gut microbiota also modifies drug response indirectly by reshaping host physiology through immune regulation, endocrine signaling, intestinal barrier function, bile acid metabolism, and modulation of hepatic drug-metabolizing enzymes [64–66]. These indirect mechanisms often influence multiple therapeutic classes simultaneously and may explain why patients with similar genetic backgrounds frequently exhibit markedly different responses to identical pharmacological interventions. Unlike direct enzymatic transformations, which involve structural modification of drug molecules, indirect mechanisms alter the biological environment in which drugs are absorbed, metabolized, distributed, and exert their therapeutic effects [5–7]. Collectively, the mechanisms described across pharmacomicrobiomic studies can be organized into three complementary categories: direct enzymatic biotransformation of drugs by microbial enzymes, modulation of host metabolic pathways through microbially derived metabolites, and alteration of intestinal barrier function that changes systemic drug exposure [67–69]. Together, these processes explain how microbial communities can increase, decrease, or redirect drug activity and provide a mechanistic framework for understanding variability in both the efficacy and toxicity of natural compounds, synthetic pharmaceuticals, and biologic therapies [49,70].

One of the principal mechanisms involves microbial regulation of intestinal barrier integrity and epithelial transport. The intestinal epithelium represents the first interface between orally administered drugs and the systemic circulation, where efficient absorption depends on a highly regulated balance between permeability, mucus composition, tight-junction integrity, and transporter activity [67]. Commensal microorganisms continuously reinforce this barrier through the production of metabolites such as short-chain fatty acids (SCFAs), particularly butyrate, acetate, and propionate, which serve as energy substrates for colonocytes while promoting tight-junction assembly, mucus secretion, and epithelial renewal [1,71]. In contrast, dysbiosis frequently disrupts epithelial integrity, increasing intestinal permeability, and facilitating systemic translocation of bacterial products including lipopolysaccharide (LPS), peptidoglycans, and flagellin [72,73]. These inflammatory changes modify the absorption kinetics of numerous orally administered drugs and may contribute to substantial interindividual variability in bioavailability [74,75]. Alterations in mucus thickness and epithelial transporter expression, including members of the ATP-binding cassette (ABC) and solute carrier (SLC) transporter families, further influence intestinal drug uptake and efflux, particularly for compounds with narrow therapeutic windows [76,77].

Beyond maintaining epithelial homeostasis, microbial metabolites function as signaling molecules that regulate numerous host pathways involved in drug metabolism. SCFAs are among the best-characterized examples. Produced through bacterial fermentation of dietary fiber by genera including *Faecalibacterium*, *Roseburia*, *Eubacterium*, and *Anaerostipes* [78,79]. SCFAs influence glucose metabolism, lipid homeostasis, intestinal immunity, and systemic inflammation through activation of G protein-coupled receptors GPR41, GPR43, and GPR109A, while also acting as histone deacetylase inhibitors that regulate epigenetic gene expression [80,81]. These effects extend beyond intestinal physiology, influencing hepatic metabolism, insulin sensitivity, and immune cell differentiation, thereby indirectly modifying the pharmacodynamics of antidiabetic agents, immunotherapies, anti-inflammatory drugs, and cardiovascular medications [82,83]. Likewise, microbial metabolism of dietary tryptophan generates indole derivatives that activate the aryl hydrocarbon receptor (AhR), promoting epithelial repair, regulating mucosal immunity, and modulating inflammatory responses that influence therapeutic outcomes across multiple disease states [80–83].

The gut microbiome also plays a central role in bile acid metabolism, another pathway increasingly recognized as a regulator of drug disposition [84]. Primary bile acids synthesized in the liver undergo extensive microbial deconjugation and transformation into secondary bile acids through the action of bacterial bile salt hydrolases and 7α-dehydroxylases [84,85]. These metabolites act as endogenous ligands for the nuclear receptors farnesoid X receptor (FXR) and Takeda G protein-coupled receptor 5 (TGR5), which regulate lipid metabolism, glucose homeostasis, intestinal barrier function, and inflammatory signaling [7,84–87]. Activation of FXR also influences the expression of hepatic and intestinal transporters together with several cytochrome P450 enzymes involved in xenobiotic metabolism [86,87]. Consequently, alterations in microbial bile acid metabolism can indirectly modify drug clearance, oral bioavailability, and therapeutic efficacy [7,84–87]. These interactions appear particularly relevant for statins, antidiabetic medications, immunomodulators, and several classes of anticancer agents whose pharmacokinetics are tightly linked to bile acid transport and enterohepatic circulation [8,86,87].

An equally important mechanism involves microbial regulation of hepatic drug-metabolizing enzymes. Although the liver remains the principal site of xenobiotic metabolism, accumulating evidence demonstrates that intestinal microorganisms influence hepatic gene expression through a continuous exchange of microbial metabolites, inflammatory mediators, and endocrine signals [88]. Germ-free animal models consistently exhibit altered expression of multiple cytochrome P450 isoforms compared with conventionally colonized animals, highlighting the dependence of hepatic metabolism on microbial colonization [47,89]. Microbial metabolites regulate several nuclear receptors, including pregnane X receptor (PXR), constitutive androstane receptor (CAR), FXR, and AhR, which collectively coordinate the transcription of CYP3A4, CYP2C9, CYP2D6, CYP1A2, and numerous phase II conjugation enzymes [90,91]. Through these pathways, dysbiosis may alter hepatic clearance, systemic drug exposure, and metabolite formation independently of host genetic polymorphisms. These observations are particularly relevant for drugs with narrow therapeutic indices such as cyclosporine A, tacrolimus, warfarin, and several chemotherapeutic agents, where relatively small alterations in CYP activity can significantly affect efficacy or toxicity [89–92].

Host immunity provides another critical intersection between microbial ecology and pharmacology. The intestinal microbiota continuously educates both innate and adaptive immune responses through interactions with epithelial cells, dendritic cells, macrophages, neutrophils, innate lymphoid cells, and T lymphocyte subsets [35,93,94]. Commensal microorganisms promote immune tolerance by stimulating regulatory T-cell differentiation and anti-inflammatory cytokines such as IL-10 while simultaneously maintaining the capacity for rapid antimicrobial defense [1,35,93,94]. Dysbiosis shifts this balance toward chronic low-grade inflammation characterized by increased production of TNF-α, IL-1β, IL-6, IL-17, and interferon-γ [1,35,93,94]. These cytokines profoundly influence drug response by modifying receptor expression, intracellular signaling cascades, transporter activity, and hepatic metabolic capacity. Chronic inflammation has been shown to suppress several CYP450 enzymes, thereby prolonging drug half-life while simultaneously reducing the efficacy of biologic therapies and immunomodulators [95]. Thus, microbiome-associated changes in systemic and intestinal inflammatory signaling may contribute to variability in therapeutic response by modifying drug-metabolizing enzyme activity, receptor signaling, immune function, and pharmacodynamic pathways.

Perhaps the most compelling demonstration of these indirect mechanisms comes from cancer immunotherapy. Unlike conventional cytotoxic drugs, immune checkpoint inhibitors rely almost entirely on host immune competence rather than direct tumor toxicity [96]. Multiple studies have demonstrated that the presence of bacterial taxa including *Akkermansia muciniphila*, *Bifidobacterium longum*, *Faecalibacterium prausnitzii*, and selected members of the Ruminococcaceae family correlates with improved responses to PD-1, PD-L1, and CTLA-4 blockade [96,97]. Rather than metabolizing the therapeutic antibodies themselves, these microorganisms enhance dendritic cell maturation, increase antigen presentation, promote CD8^+^ T-cell activation, and augment interferon-γ signaling within the tumor microenvironment, thereby improving antitumor immunity [9–11]. Conversely, depletion of microbial diversity through antibiotic exposure or profound dysbiosis is consistently associated with reduced response rates, shorter progression-free survival, and diminished overall survival across multiple malignancies [97,98]. These findings illustrate that microbial influences on pharmacology extend well beyond drug metabolism and encompass the regulation of entire physiological systems that ultimately determine therapeutic success.

Collectively, these indirect mechanisms emphasize that the microbiome influences drug efficacy through far more than enzymatic biotransformation alone. By regulating epithelial barrier function, microbial metabolite production, nuclear receptor signaling, immune homeostasis, inflammatory responses, and hepatic xenobiotic metabolism, intestinal microorganisms create a physiological landscape that shapes virtually every aspect of pharmacokinetics and pharmacodynamics. Appreciating these interconnected pathways is fundamental for developing microbiome-informed therapeutic strategies and provides the mechanistic foundation for precision medicine approaches that integrate microbial function alongside host genetics, clinical variables, and environmental exposures.

## 4. Impact of the Microbiome on Drug Efficacy

### 4.1. Oncology: The Microbiome as a Determinant of Cancer Therapeutic Response

Among all therapeutic areas, oncology has provided the most compelling evidence that the human microbiome functions as a critical determinant of drug efficacy rather than merely a passive modifier of host physiology. Over the past decade, numerous preclinical and clinical studies have demonstrated that the composition and functional capacity of the gut microbiome influence therapeutic outcomes across virtually every major class of anticancer agents, including conventional chemotherapy, immune checkpoint inhibitors (ICIs), targeted therapies, hormone therapies, and cellular immunotherapies [5,9,11]. These discoveries have transformed the microbiome from an observational biomarker into a mechanistic component of cancer pharmacology and have established pharmacomicrobiomics as an emerging pillar of precision oncology [5,9,11].

Unlike pharmacogenomics, which primarily explains inherited variation in drug metabolism and target sensitivity, the microbiome introduces a dynamic and potentially modifiable layer of biological variability. Individual differences in microbial diversity, metabolic activity, and microbial gene content have repeatedly been associated with substantial variation in treatment efficacy, immune activation, toxicity, and long-term survival [1,3,35,42]. Importantly, these differences are influenced by diet, antibiotic exposure, disease status, age, environmental factors, and previous medications, suggesting that therapeutic response is shaped not only by tumor biology and host genetics but also by the ecological state of the intestinal microbiota [7,8,99]. Oncology is discussed in greater depth in this review than other therapeutic areas because it currently provides some of the strongest clinical evidence linking gut microbial composition to therapeutic response; however, emerging evidence in cardiovascular, metabolic, neurological, and psychiatric pharmacology demonstrates that pharmacomicrobiomic mechanisms extend well beyond cancer.

Current evidence indicates that the microbiome influences anticancer therapy through three interconnected mechanisms. First, microorganisms directly metabolize chemotherapeutic agents through bacterial enzymes capable of activating, inactivating, or toxifying drugs before systemic absorption or during enterohepatic recirculation [100]. Second, microbial metabolites reshape host immunity by regulating dendritic cell maturation, T-cell differentiation, cytokine production, and inflammatory signaling pathways that determine antitumor immune responses [101]. Third, the microbiome remodels the tumor microenvironment through effects on angiogenesis, epithelial barrier integrity, oxidative stress, and immune-cell infiltration, thereby influencing tumor sensitivity to both cytotoxic and immunological therapies [9,102,103] (Figure 2).

#### 4.1.1. Immune Checkpoint Inhibitors

Perhaps no therapeutic class illustrates the importance of the microbiome more convincingly than immune checkpoint inhibitors targeting programmed cell death protein 1 (PD-1), programmed death ligand 1 (PD-L1), and cytotoxic T-lymphocyte-associated protein 4 (CTLA-4). Although these therapies have revolutionized the treatment of melanoma, non-small cell lung cancer, renal cell carcinoma, colorectal cancer, hepatocellular carcinoma, and several other malignancies, only a subset of patients achieves durable clinical responses. The microbiome has emerged as one of the strongest predictors of this variability [4,10,97]. Commercially available monoclonal antibodies, particularly the anti-PD-1 agents; pembrolizumab and nivolumab, exemplify the clinical relevance of microbiome-mediated immune regulation [104]. Clinical studies consistently demonstrate that patients with greater gut microbial diversity and enrichment of taxa such as *Akkermansia muciniphila* and *Bifidobacterium* spp. exhibit improved responses to PD-1 blockade, whereas dysbiosis and antibiotic-induced disruption of the intestinal microbiome are associated with impaired antitumor immunity and poorer clinical outcomes [10,97,105].

Building upon these clinical observations, multiple independent studies have identified additional microbial signatures associated with favorable immunotherapy responses, including *Faecalibacterium prausnitzii*, *Ruminococcus* spp., *Alistipes shahii*, *Bacteroides fragilis*, and *Enterococcus hirae* [10,106]. Rather than directly interacting with immune checkpoint inhibitors, these microorganisms enhance antitumor immunity by promoting dendritic cell maturation, improving antigen presentation, increasing CD8^+^ cytotoxic T-cell infiltration, stimulating interferon-γ production, and expanding memory T-cell populations [103]. Collectively, these immunomodulatory effects amplify the therapeutic activity of PD-1- and CTLA-4-targeted therapies (Figure 2). Among these microorganisms, *Akkermansia muciniphila* has become one of the most extensively studied. Oral administration of *A. muciniphila* restored responsiveness to PD-1 blockade in germ-free or antibiotic-treated mice by increasing IL-12 production and promoting recruitment of CCR9^+^CXCR3^+^CD4^+^ T lymphocytes into the tumor microenvironment [107]. Similar associations have subsequently been reported in patients with lung cancer, melanoma, and renal cell carcinoma, where enrichment of *A. muciniphila* correlates with longer progression-free and overall survival [9,10].

Conversely, disruption of microbial diversity frequently compromises immunotherapy efficacy. Numerous retrospective and prospective studies have demonstrated that exposure to broad-spectrum antibiotics immediately before or during immune checkpoint inhibitor therapy significantly reduces objective response rates, progression-free survival, and overall survival across multiple malignancies [10,108]. Similar, although less consistent effects have been reported for proton pump inhibitors and corticosteroids, likely reflecting their capacity to alter microbial composition and reduce microbial diversity [109,110]. These observations emphasize that concomitant medications capable of modifying the microbiome should be carefully considered when interpreting immunotherapy outcomes.

#### 4.1.2. Chemotherapy

The microbiome also exerts profound effects on conventional cytotoxic chemotherapy. Unlike immunotherapy, where immune modulation predominates, chemotherapy is influenced through both direct microbial metabolism and indirect regulation of inflammation, oxidative stress, epithelial repair, and immune activation [111]. Cyclophosphamide provides one of the earliest demonstrations of microbiome-dependent chemotherapy. Treatment with cyclophosphamide transiently increases intestinal permeability, allowing selected Gram-positive bacteria, including *Enterococcus hirae* and *Lactobacillus johnsonii*, to translocate into secondary lymphoid organs [112]. Rather than representing a pathological complication, this bacterial translocation stimulates differentiation of pathogenic Th17 and memory Th1 immune responses that substantially enhance antitumor immunity [113]. Elimination of these microorganisms through antibiotic treatment markedly reduces cyclophosphamide efficacy in experimental models, highlighting the microbiome as an essential component of therapeutic success [112].

Among chemotherapeutic agents, irinotecan represents one of the best-characterized examples of microbiome-mediated toxicity. Following hepatic glucuronidation of its active metabolite SN-38, microbial β-glucuronidases regenerate toxic SN-38 within the intestinal lumen, causing epithelial injury, mucositis, and severe delayed diarrhea [59]. Because this toxicity originates from bacterial rather than host enzymes, considerable effort has focused on developing selective microbial β-glucuronidase inhibitors capable of preventing gastrointestinal toxicity without interfering with host glucuronidation pathways [56,57]. Additional examples continued to emerge. Several members of the Gammaproteobacteria, including *Escherichia coli*, express cytidine deaminase capable of converting gemcitabine into its inactive metabolite, thereby promoting chemoresistance [114]. Likewise, alterations in microbial diversity have been associated with differential responses to oxaliplatin, 5-fluorouracil, methotrexate, and platinum-based regimens through mechanisms involving reactive oxygen species generation, inflammatory cytokine production, and modulation of DNA damage responses [2,9,111].

Beyond influencing initial therapeutic response, accumulating evidence indicates that the microbiome contributes to acquired drug resistance. Resistance mechanisms extend beyond direct drug metabolism and involve complex interactions between microbial metabolites, host immunity, and tumor biology. Short-chain fatty acids, secondary bile acids, polyamines, and tryptophan-derived metabolites regulate epigenetic modifications, cancer cell metabolism, epithelial–mesenchymal transition, and immune suppression, collectively creating a tumor microenvironment that favors resistance to chemotherapy, targeted therapies, and immunotherapy [2,9,111]. Recent molecular studies have further demonstrated that microbial signaling can activate oncogenic pathways including PI3K/AKT, Wnt/β-catenin, NF-κB, STAT3, and MAPK, promoting tumor proliferation while reducing susceptibility to apoptosis induced by anticancer agents [115]. At the same time, microbial modulation of macrophage polarization, regulatory T-cell expansion, and myeloid-derived suppressor cell recruitment suppresses effective antitumor immunity and diminishes responsiveness to immunotherapy [2,9,111,115,116].

#### 4.1.3. Oncolytic Viruses

Oncolytic viruses (OVs) combine direct tumor lysis with stimulation of antitumor immunity and have achieved clinical approval for talimogene laherparepvec (T-VEC) in melanoma and teserpaturev (G47Δ, Delytact) in malignant glioma, alongside numerous adenoviral, herpesviral, vaccinia, reovirus, and vesicular stomatitis virus-based platforms in development [117]. Accumulating evidence indicates that the gut microbiome represents an additional determinant of OV efficacy: microbial composition influences viral replication, innate immune activation, antigen presentation, and the generation of durable adaptive antitumor responses by shaping dendritic cell maturation, interferon signaling, and lymphocyte activation [2,115,116,118].

Three mechanistic examples illustrate this dependence. First, depletion of the intestinal microbiota enhanced VSVΔ 51 replication and antitumor CD8^+^ T-cell activity in hepato-cellular carcinoma models, and restoration of Lactobacillus acidophilus re-established gut barrier integrity and potentiated multiple OV platforms, including VSVΔ 51, HSV-1, and vaccinia virus [119]. Second, the microbial metabolite butyrate increased infectivity and immunogenicity of a p53-expressing telomerase-specific oncolytic adenovirus in colorectal cancer models, acting through coxsackievirus/adenovirus receptor upregulation, cGAS-STING activation, and CD8^+^ T-cell recruitment [120]. Third, oral (rather than intratumoral) administration of the oncolytic reovirus RC402 reshaped the gut microbiome and generated systemic antitumor immunity through Peyer’s-patch immune activation; depletion of the gut microbiota abolished this effect, and combining oral reovirus with PD-1 and CTLA-4 blockade produced durable tumor regression in preclinical models [121].

Naturally occurring differences in gut microbial composition have also been associated with responsiveness to the oncolytic adenovirus Delta-24-RGDOX in malignant glioma, supporting combination strategies that pair virotherapy with microbiome-directed interventions [122]. Although these findings provide compelling mechanistic rationale, microbiome-dependent modulation of oncolytic virotherapy remains predominantly supported by preclinical models. Whether microbial signatures or microbiome-directed interventions can reproducibly predict or improve OV response in patients remains to be established in prospective clinical studies.

#### 4.1.4. Microbiome-Based Therapeutic Strategies

Recognition of the microbiome as a determinant of cancer therapy has stimulated intense interest in microbiome-directed interventions aimed at improving therapeutic efficacy while minimizing toxicity. Among these approaches, fecal microbiota transplantation (FMT) has generated the strongest clinical evidence. Early clinical trials demonstrated that transferring fecal microbiota from patients who responded to immune checkpoint inhibitors into refractory melanoma patients restored sensitivity to anti-PD-1 therapy in a subset of recipients [123,124]. These studies provided the first direct demonstration that manipulating the microbiome can alter responsiveness to cancer immunotherapy in humans [125].

Additional strategies include dietary interventions designed to increase microbial diversity, administration of prebiotics and probiotics, selective microbial enzyme inhibitors, postbiotics, bacteriophage therapy, and engineered probiotic platforms capable of delivering therapeutic molecules directly within the gastrointestinal tract [126,127]. Advances in synthetic biology are now enabling the development of programmable bacterial systems capable of sensing tumor-specific signals, producing immunomodulatory compounds, and enhancing localized drug delivery [11,12,128,129]. Although many of these approaches remain experimental, they illustrate the transition from observational microbiome research toward therapeutic manipulation of microbial ecosystems [11,12,128,129] (Figure 2).

Collectively, these discoveries have fundamentally altered our understanding of cancer pharmacology. The microbiome is no longer viewed simply as a modifier of drug metabolism but rather as an active participant in therapeutic response, capable of influencing efficacy, toxicity, immune activation, and the evolution of drug resistance. Integrating microbiota profiling into oncology practice may therefore improve patient stratification, predict therapeutic outcomes, and facilitate the development of personalized treatment strategies that combine conventional anticancer agents with microbiome-targeted interventions. This evolving paradigm represents one of the clearest examples of how pharmacomicrobiomics is reshaping precision medicine and provides a framework that is increasingly applicable across other therapeutic disciplines (Figure 2).

An emerging concept in this area is that of “autobiotic” approaches, which seek to support or restore an individual’s endogenous microbial ecosystem rather than primarily introducing standardized exogenous microbial strains. The term has been used for strategies combining microbiota-supporting components, including prebiotic and postbiotic interventions, and in some contexts for individualized microbiota-derived approaches. Conceptually, autobiotic strategies are relevant to precision pharmacomicrobiomics because they emphasize the patient’s baseline microbial ecology and functional capacity rather than a one-size-fits-all formulation. However, terminology in this space remains inconsistent, the concept is not yet standardized as a therapeutic category, and robust clinical evidence demonstrating superiority over established prebiotic, probiotic, synbiotic, or postbiotic interventions remains limited. Accordingly, autobiotics should currently be regarded as an emerging rather than an established microbiome-directed strategy.

### 4.2. Neurological Disorders

The bidirectional communication between the gastrointestinal microbiota and the central nervous system, collectively known as the microbiota–gut–brain axis, has emerged as one of the most rapidly expanding areas of pharmacomicrobiomics [54,55,130]. This complex network integrates neural, endocrine, metabolic, and immune pathways through continuous interactions among intestinal microorganisms, the enteric nervous system, the vagus nerve, immune mediators, and the hypothalamic–pituitary–adrenal axis (Figure 3) [54,55,130,131]. Beyond its established role in neurological development and neuroinflammation, growing evidence indicates that the gut microbiome significantly influences the pharmacokinetics and pharmacodynamics of numerous neurological drugs, contributing to the remarkable interindividual variability observed in patients with Parkinson’s disease, Alzheimer’s disease, epilepsy, depression, and other neurodegenerative disorders [5,42,54,55,130,131].

Among all neurological medications, levodopa (L-DOPA) represents the best-characterized example of microbiome-dependent drug metabolism [53]. As the gold standard treatment for Parkinson’s disease, levodopa must reach the central nervous system before conversion into dopamine. However, this process is frequently compromised by intestinal microorganisms. *Enterococcus faecalis* expresses a pyridoxal phosphate-dependent tyrosine decarboxylase capable of converting levodopa into dopamine within the intestinal lumen before absorption [53,54]. Since dopamine cannot efficiently cross the blood–brain barrier, this premature conversion substantially reduces levodopa bioavailability. The situation becomes even more complex because *Eggerthella lenta* subsequently converts dopamine into *m*-tyramine through a molybdenum-dependent dehydroxylase, further decreasing therapeutic efficacy [53,54]. These sequential microbial transformations explain why patients receiving identical doses frequently exhibit markedly different clinical responses despite comparable host pharmacogenetic profiles (Figure 3) [42,53,54].

The implications of these discoveries extend beyond levodopa metabolism. Alterations in gut microbial composition have consistently been associated with Parkinson’s disease progression, with reductions in SCFA-producing bacteria including *Faecalibacterium*, *Roseburia*, and *Prevotella*, accompanied by expansion of opportunistic pathogens such as Enterobacteriaceae [54]. Reduced production of butyrate compromises intestinal barrier integrity and promotes systemic inflammation, facilitating α-synuclein aggregation and neuroinflammation that may accelerate disease progression [1,54,55]. Consequently, therapeutic strategies targeting microbial communities may simultaneously improve drug bioavailability and modify disease progression (Figure 3) [1,54,55].

The microbiome also influences the metabolism of antiepileptic drugs, opioids, and anesthetic agents, although these mechanisms remain less thoroughly characterized [42,63]. Several microbial metabolites alter blood–brain barrier permeability, neurotransmitter synthesis, and neuroimmune signaling through modulation of tryptophan metabolism, serotonin biosynthesis, γ-aminobutyric acid (GABA) production, and glutamatergic neuro-transmission [54,132]. Bacterial production of indole derivatives, kynurenine metabolites, and SCFAs further regulates microglial activation and neuroinflammatory pathways that influence therapeutic response in multiple neurological disorders [42,130].

As microbiota profiling becomes increasingly integrated into neurological research, personalized modulation of intestinal microbial communities may eventually complement conventional pharmacotherapy [54]. Probiotics, dietary interventions, microbial enzyme inhibitors, and fecal microbiota transplantation are currently under investigation as strategies to enhance therapeutic response while simultaneously reducing neuroinflammation and disease progression [54].

Overall, however, most evidence linking the gut microbiome to neurological drug response remains preclinical or derived from small human cohorts. Levodopa–microbiome interactions are comparatively well-characterized mechanistically, but prospective studies directly testing microbiome-based interventions to improve neurological drug response remain scarce, and differences in sampling, sequencing platforms, and patient populations limit cross-study comparison.

### 4.3. Psychiatric Disorders

Growing evidence indicates that variability in the efficacy and adverse effects of psychiatric medications cannot be explained exclusively by host genetics or central nervous system pharmacology [133]. The gut microbiota has emerged as an additional determinant of therapeutic response through its extensive communication with the brain via the microbiota–gut–brain axis [55,134]. This bidirectional network integrates neural, endocrine, metabolic, and immune pathways through vagal signaling, microbial metabolites, neuroactive compounds, and cytokine-mediated communication [130–132,135]. Consequently, alterations in microbial composition influence neurotransmitter availability, blood–brain barrier integrity, neuroinflammation, and neuronal plasticity, all of which affect the pharmacodynamics of antidepressants, antipsychotics, anxiolytics, and mood stabilizers [5,42,55,134] (Figure 3).

The intestinal microbiota contributes directly to the synthesis and metabolism of numerous neuroactive molecules, including serotonin, γ-aminobutyric acid (GABA), dopamine, norepinephrine, acetylcholine, and histamine [136]. Although most serotonin produced in the intestine does not cross the blood–brain barrier, microbial regulation of tryptophan metabolism profoundly influences central serotonergic signaling through the kynurenine pathway and production of indole derivatives that activate the aryl hydrocarbon receptor (AhR) [137]. Likewise, SCFAs modulate microglial maturation, neurogenesis, synaptic plasticity, and epigenetic regulation through inhibition of histone deacetylases, linking microbial metabolism to central nervous system function [80,134].

Antidepressants represent one of the clearest examples of bidirectional microbiome–drug interactions. Selective serotonin reuptake inhibitors (SSRIs), particularly fluoxetine and sertraline, exhibit intrinsic antimicrobial activity capable of altering microbial diversity independently of their effects on serotonin transporters [138]. These alterations frequently involve reductions in *Bacteroides*, *Clostridium*, and other anaerobic taxa while increasing the relative abundance of opportunistic microorganisms [139,140]. Conversely, baseline microbiota composition has been associated with antidepressant responsiveness, suggesting that microbial diversity and metabolite production may partially explain why approximately one-third of patients fail first-line antidepressant therapy [5,8,141].

Antipsychotic medications similarly interact with the microbiome. Second-generation antipsychotics, including olanzapine and risperidone, consistently induce microbial dysbiosis characterized by reductions in beneficial SCFA-producing bacteria and expansion of inflammatory taxa [142,143]. These microbial alterations contribute to weight gain, insulin resistance, dyslipidemia, and chronic inflammation that frequently accompany long-term antipsychotic treatment [142,143]. Experimental studies have demonstrated that depletion of the gut microbiota attenuates olanzapine-induced metabolic dysfunction, providing compelling evidence that the microbiome contributes directly to these adverse effects rather than simply reflecting them [5,144].

Although pharmacomicrobiomics in psychiatry remains in its early stages, the convergence of microbiome research, neuroscience, and psychopharmacology suggests that future treatment strategies may incorporate microbial biomarkers to predict therapeutic response while combining conventional medications with dietary interventions, psychobiotics, or microbiome-modulating therapies designed to enhance treatment efficacy and reduce metabolic toxicity (Figure 3).

As in neurology, the evidence base for psychiatric pharmacomicrobiomics is predominantly associative and derived from observational cohorts and animal models rather than randomized interventional trials. Antidepressant- and antipsychotic-microbiome associations are biologically plausible and increasingly reproducible across studies, but causal direction, and whether microbiome-informed dosing or adjunctive microbiome-targeted therapy meaningfully improves psychiatric outcomes, has not yet been established in adequately powered clinical trials.

### 4.4. Cardiovascular Medicine

Although cardiovascular pharmacology has traditionally focused on host genetics, hepatic metabolism, and renal clearance, accumulating evidence demonstrates that the gut microbiome substantially contributes to variability in cardiovascular drug response [145]. This influence extends beyond direct drug metabolism and encompasses microbial regulation of bile acid homeostasis, lipid metabolism, inflammatory signaling, platelet activation, endothelial function, and production of bioactive metabolites such as trimethylamine N-oxide (TMAO), secondary bile acids, and short-chain fatty acids [47,62,145] (Figure 4).

The classical example of microbiome-mediated cardiovascular pharmacology involves digoxin, one of the earliest drugs shown to undergo direct bacterial metabolism [52]. Approximately 10% of patients receiving digoxin harbor strains of *Eggerthella lenta* expressing the cardiac glycoside reductase (cgr) operon, which catalyzes reduction in digoxin into inactive dihydrodigoxin before systemic absorption [52,146]. Importantly, the presence of *E. lenta* alone does not predict drug inactivation. Rather, expression of the cgr operon is strongly influenced by dietary arginine availability, illustrating how host diet, microbial gene regulation, and pharmacology interact to determine therapeutic efficacy [52,146,147]. This discovery represents one of the clearest demonstrations that microbial gene expression, rather than microbial abundance alone, can determine clinical response.

Microbial regulation of bile acid metabolism also influences several cardiovascular medications [85]. Intestinal microorganisms expressing bile salt hydrolases and 7α-dehydroxylases modify primary bile acids into secondary bile acids that activate FXR and TGR5 signaling pathways, thereby regulating cholesterol metabolism, glucose homeostasis, hepatic transporter expression, and CYP450 activity [7,84,148]. These mechanisms may contribute to interindividual variability observed with statins, ezetimibe, fibrates, and bile acid sequestrants, although clinical validation remains ongoing. Microbial metabolites further influence cardiovascular disease independently of drug metabolism. Conversion of dietary choline, phosphatidylcholine, and L-carnitine into trimethylamine (TMA), subsequently oxidized to trimethylamine N-oxide (TMAO) by hepatic flavin monooxygenases, has been associated with accelerated atherosclerosis, thrombosis, endothelial dysfunction, and adverse cardiovascular outcomes [62,149]. Because several cardiovascular therapies target these same pathological pathways, modulation of microbial TMA production has emerged as a promising adjunctive therapeutic strategy [150].

Recent investigations also suggest reciprocal interactions between cardiovascular medications and the microbiome [62,145]. Statins, antihypertensive agents, angiotensin-converting enzyme inhibitors, angiotensin receptor blockers, and β-blockers all induce measurable alterations in microbial diversity and metabolic activity, potentially contributing to their pleiotropic anti-inflammatory effects [145,151,152]. Although the clinical significance of these microbial alterations remains under investigation, they further illustrate the bidirectional relationship between pharmaceuticals and intestinal microbial ecology [8,145,151,152].

Taken together, cardiovascular pharmacomicrobiomic associations are supported by consistent mechanistic and animal data, particularly for TMAO- and bile-acid-related pathways, but clinical evidence that microbiome-directed interventions improve cardiovascular drug response remains limited to small or short-term trials. Larger, longer-term interventional studies are needed before microbial biomarkers can guide cardiovascular therapy in practice.

### 4.5. Diabetes and Metabolic Diseases

Among commonly prescribed medications, metformin has become one of the best examples demonstrating that therapeutic efficacy may depend partly on microbiome modulation rather than solely on direct host pharmacology. Although metformin has been prescribed for more than six decades, only recently has it become apparent that a significant proportion of its glucose-lowering activity results from alterations in intestinal microbial composition and function rather than direct hepatic inhibition of gluconeogenesis [5,153]. Treatment with metformin consistently increases the abundance of *Akkermansia muciniphila*, *Bifidobacterium*, *Escherichia*, and multiple SCFA-producing bacteria while decreasing several taxa associated with metabolic inflammation [5,153,154]. These microbial shifts enhance production of butyrate and propionate, strengthen epithelial barrier integrity, reduce metabolic endotoxemia, and improve insulin sensitivity through activation of GPR41 and GPR43 signaling pathways [155–157]. Increased production of secondary bile acids further activates FXR and TGR5 receptors, contributing to improved glucose homeostasis and enhanced secretion of glucagon-like peptide-1 (GLP-1) [86,87]. Consequently, part of metformin’s therapeutic benefit appears to result from remodeling the intestinal ecosystem rather than acting exclusively on host tissues (Figure 4).

Microbial metabolism also contributes to the efficacy of several naturally occurring antidiabetic compounds. Berberine, for example, exhibits poor intestinal absorption yet demonstrates substantial glucose-lowering activity after microbial conversion into more bioavailable metabolites [158]. Similar microbiome-dependent activation has been reported for ginsenosides, ellagitannins, flavonoids, and numerous polyphenolic compounds, highlighting the importance of microbial biotransformation in both conventional pharmacology and phytotherapy [12,159]. Conversely, metabolic diseases themselves profoundly alter microbial ecology (Figure 4). Obesity and type 2 diabetes are frequently characterized by reduced microbial diversity, depletion of butyrate-producing bacteria, expansion of pro-inflammatory taxa, increased intestinal permeability, and chronic low-grade inflammation [160,161] (Figure 4). These alterations influence not only disease progression but also therapeutic response to antidiabetic medications, suggesting that baseline microbiota composition may eventually become a predictive biomarker for treatment selection [1,8] (Figure 4).

Collectively, these findings support a paradigm in which successful management of metabolic disorders requires consideration of the host–microbiome ecosystem rather than host metabolism alone. Future therapeutic strategies are therefore likely to combine conventional glucose-lowering medications with microbiome-directed interventions designed to restore microbial diversity, enhance SCFA production, and improve metabolic resilience (Figure 4).

Metformin remains the best-characterized example of microbiome-dependent metabolic drug response, with converging mechanistic and human data; evidence for other antidiabetic and metabolic agents is comparatively preliminary and often confounded by diet, adiposity, and disease duration. As in other therapeutic areas, microbiome-based metabolic biomarkers require prospective validation before routine clinical use.

### 4.6. Bidirectional Interactions Between Natural Products and the Gut Microbiome

Microbial transformation of natural products should not be viewed exclusively as an activation process. Depending on the compound, microbial species, and enzymatic pathway involved, gut microorganisms may (i) activate poorly active precursors, (ii) inactivate biologically active parent compounds, (iii) generate structurally distinct metabolites with new pharmacological activities, (iv) increase or decrease intestinal absorption and systemic bioavailability, and (v) generate metabolites with altered toxicity profiles. Consequently, the same natural product may produce markedly different biological effects among individuals with different microbial metabolic capacities. Conversely, phytochemicals and their metabolites can selectively inhibit or promote microbial taxa, modify SCFA production, alter bile-acid pools, influence barrier integrity, and reshape inflammatory signaling, establishing a reciprocal pharmacological relationship. The examples below illustrate each of these outcomes.

Curcumin, a polyphenol derived from turmeric, illustrates this reciprocal relationship particularly well. Despite well-documented anti-inflammatory, antioxidant, and immunomodulatory activities, curcumin has inherently poor intrinsic bioavailability [162,163], and the gut microbiota substantially determine its systemic exposure and biological activity, with antibiotic-mediated depletion of the microbiota altering curcumin metabolism in animal models [164]. Beyond its own biotransformation, curcumin also modulates intestinal epithelial barrier integrity by inhibiting endoplasmic-reticulum stress and epithelial apoptosis [165]. Related dietary flavonoids, quercetin, and catechins similarly depend on microbial metabolism for their systemic bioavailability and contribute to protection against metabolic disease [166,167], while microbial transformation of flavonoids more broadly shapes host lipid metabolism [168]. Resveratrol undergoes comparable microbial biotransformation, with gut-derived metabolites such as dihydroresveratrol and lunularin contributing substantially to its overall biological activity [169,170]. Collectively, these compound-specific examples reinforce the general principle that the pharmacological effects of natural products are frequently mediated, and can be substantially modified, by the gut microbiota [171].

Natural products provide one of the clearest examples of the reciprocal relationship between therapeutic compounds and the human microbiome. Unlike many synthetic drugs, numerous phytochemicals possess limited oral bioavailability and rely on microbial metabolism to generate bioactive metabolites capable of exerting systemic effects [61,172]. Conversely, these same compounds actively remodel the intestinal microbiota by promoting beneficial microbial populations, suppressing opportunistic pathogens, and altering microbial metabolic activity [172,173]. Consequently, the therapeutic efficacy of many plant-derived compounds depends not only on their intrinsic chemical properties but also on the composition and functional capacity of the host microbiome [61,173,174] (Figure 5).

This bidirectional interaction is particularly evident for dietary polyphenols, which are abundant in fruits, vegetables, tea, cocoa, coffee, and medicinal plants [175]. Owing to their complex glycosylated structures, most polyphenols escape absorption in the upper gastroin-testinal tract and reach the colon largely intact [175]. There, bacterial glycosidases, esterases, decarboxylases, demethylases, and dehydroxylases convert them into smaller phenolic metabolites with greater bioavailability and biological activity [7,176]. Consequently, many of the physiological effects traditionally attributed to dietary polyphenols are mediated by microbiome-derived metabolites rather than by the parent compounds themselves [177]. Interindividual differences in microbial composition therefore contribute substantially to variability in pharmacological response, emphasizing that microbial functional capacity is often a stronger determinant of efficacy than dietary intake alone.

Several phytochemicals exemplify this microbiome-dependent metabolism. Ellagitannins, abundant in pomegranates, walnuts, berries, and other fruits, are transformed into urolithins, particularly urolithin A, a metabolite with anti-inflammatory, antioxidant, mitochondrial protective, and anti-aging properties [178]. However, only individuals harboring specific microbial communities efficiently produce urolithins, giving rise to distinct metabolic phenotypes that influence therapeutic responsiveness [12]. Likewise, ginsenosides require sequential microbial deglycosylation before conversion into compound K, the metabolite responsible for many of the neuroprotective, anticancer, antidiabetic, and immunomodulatory effects attributed to *Panax* species [179,180]. Similar microbiome-dependent activation has been reported for daidzein, which is converted into the more biologically active metabolite equol, and for glucosinolates, whose microbial transformation into isothiocyanates contributes to their chemopreventive activity [12,181].

Berberine further illustrates the reciprocal nature of these interactions. Although berberine exhibits poor oral bioavailability, intestinal microorganisms convert it into dihydroberberine, a more lipophilic intermediate that is absorbed more efficiently before being oxidized back to berberine within host tissues [182,183]. At the same time, berberine reshapes the intestinal microbiota by enriching beneficial taxa such as *Akkermansia muciniphila*, *Bifidobacterium*, and several short-chain fatty acid (SCFA)-producing bacteria while reducing microorganisms associated with metabolic inflammation [184,185]. These microbial shifts improve epithelial barrier integrity, regulate bile acid metabolism, decrease systemic inflammation, and enhance glucose homeostasis, demonstrating that part of berberine’s therapeutic activity results from remodeling the intestinal ecosystem rather than direct interaction with host targets alone [12,184].

As with the synthetic-drug classes discussed above, the strength of evidence for natural-product–microbiome interactions varies considerably by compound. Mechanistic and pharmacokinetic data for urolithins, equol, compound K, and dihydroberberine are well supported by in vitro and animal studies, whereas evidence that microbiota profiling or modulation improves clinical outcomes for botanical therapeutics is still largely preliminary and based on small human studies. Standardized product characterization combined with functional microbiome and pharmacokinetic profiling will be required to translate these findings into personalized botanical dosing strategies.

## 5. Future Perspectives and Challenges

The rapid expansion of pharmacomicrobiomics has fundamentally changed our understanding of interindividual variability in drug response. While host genetics, age, sex, organ function, and environmental exposures remain major determinants of pharmacokinetics and pharmacodynamics, it is increasingly evident that the microbiome constitutes an additional and highly dynamic layer of biological regulation. Unlike the host genome, microbial communities can be intentionally modified through diet, pharmacological interventions, microbial therapeutics, or lifestyle changes, making the microbiome an attractive target for precision medicine. Future therapeutic strategies will likely integrate host genetics with microbial composition and function to better predict treatment efficacy, minimize adverse drug reactions, and optimize individualized therapies [6,7].

A major challenge moving forward is shifting from descriptive microbial profiling toward functional characterization of microbial activity. Advances in shotgun metagenomics, meta transcriptomics, metabolomics, meta proteomics, culturomics, and single-cell sequencing now enable identification of microbial genes, enzymes, and metabolic pathways directly involved in drug metabolism rather than simply describing taxonomic composition. This transition from “who is there” to “what they are doing” is expected to substantially improve prediction of drug efficacy and toxicity by identifying microbial biomarkers associated with therapeutic response [6,7,186]. Integrating these datasets through systems biology approaches will further enhance our understanding of host–microbiome–drug interactions and facilitate the discovery of novel therapeutic targets.

Emerging technologies are also transforming experimental models used to investigate microbiome–drug interactions. Organ-on-a-chip platforms, micro physiological systems, and physiologically based pharmacokinetic (PBPK) modeling provide increasingly sophisticated alternatives to conventional in vitro and animal models by recreating dynamic interactions among intestinal epithelium, microbial communities, immune cells, and host tissues [7,15,16,93,187]. These platforms enable real-time evaluation of microbial metabolism, drug absorption, and tissue-specific responses while reducing reliance on animal experimentation [39,188,189]. Although challenges remain regarding long-term microbial stability, oxygen gradients, and biomaterial compatibility [190], continued refinement of these models is expected to accelerate microbiome-informed drug development and improve translational predictability [191].

The microbiome itself is rapidly emerging as a therapeutic target. Rather than broadly eliminating microbial communities with antibiotics, current strategies seek to selectively modulate microbial function while preserving ecological stability. Approaches under investigation include precision probiotics, prebiotics, postbiotics, fecal microbiota transplantation (FMT), bacteriophage therapy, microbial enzyme inhibitors, engineered microbial consortia, and CRISPR-based microbiome editing [3,12,15,74,187]. Recent outburst of personalized probiotic formulations illustrates this shift toward targeted microbial therapeutics. Recent clinical studies have demonstrated that multi-strain formulations such as Consti-Biome and Sensi-Biome improve constipation-related symptoms, abdominal pain, urgency, and stool consistency while promoting favorable shifts in microbial composition, suggesting that individualized microbial interventions may complement conventional pharmacological treatments [60,192,193]. Although these findings are encouraging, larger randomized clinical trials are needed to establish standardized formulations and identify the patient’s populations most likely to benefit from microbiome-targeted therapies.

Artificial intelligence (AI) and machine learning are expected to become central components of pharmacomicrobiomics by integrating multidimensional datasets that include microbial composition, functional genomics, metabolomics, clinical variables, dietary habits, medication history, and host genetics. These computational approaches have the potential to identify complex interaction networks that are difficult to detect using conventional statistical methods, enabling prediction of therapeutic response, optimization of dosing regimens, early identification of patients at risk for adverse drug reactions, and development of microbiome-informed clinical decision support systems. Ultimately, combining host genomic information with microbial functional profiles may provide a more comprehensive framework for precision medicine than either approach independently.

Despite remarkable progress, several barriers continue to limit clinical translation. Considerable variability exists in sample collection, sequencing methodologies, bioinformatic pipelines, and analytical approaches, making comparisons across studies challenging. Future integration of microbiome science into drug development will require standardized sampling procedures, sequencing workflows, bioinformatic pipelines, causal validation studies, and regulatory frameworks capable of evaluating microbiome-derived biomarkers and therapeutic interventions. Likewise, metagenomic and metabolomic approaches, organoid and gut-on-chip models, and AI-driven computational modeling are expected to play central roles in predicting microbiome-mediated drug responses and translating mechanistic discoveries into clinically actionable strategies [16,42,93,194,195]. In addition, microbiota composition is influenced by numerous confounding variables, including diet, geography, ethnicity, age, medication use, circadian rhythms, and disease status, that must be carefully controlled in both observational and interventional studies [196,197]. Establishing standardized protocols, longitudinal cohort studies, mechanistic experimental models, and large multicenter clinical trials will be essential to distinguish causal microbiome–drug interactions from associative observations and facilitate regulatory acceptance of microbiome-based biomarkers.

Collectively, the evidence reviewed throughout this manuscript demonstrates that the microbiome is no longer viewed simply as a collection of commensal microorganisms but as a dynamic metabolic, immunological, and pharmacological organ that profoundly influences the efficacy and safety of both synthetic drugs and natural products. Incorporating microbiome-informed strategies into drug discovery, clinical trial design, and routine patient care has the potential to redefine pharmacology beyond the traditional host-centered paradigm. Continued integration of microbiome science with systems biology, computational modeling, and precision medicine will be essential for developing safer therapies, improving therapeutic outcomes, and advancing the next generation of personalized healthcare.

## 6. Limitations of the Review

This narrative review has several limitations. First, because the literature was synthesized narratively rather than through a systematic review and meta-analysis, the selection of studies may be subject to publication and selection bias, and no formal quantitative assessment of study quality or effect size was performed. Second, the evidence base is heterogeneous across therapeutic areas. Oncology and selected drugs such as metformin, digoxin, levodopa, and irinotecan have comparatively well-characterized microbiome interactions, whereas evidence for many other therapeutic classes and natural products remains predominantly observational, preclinical, or mechanistic. Third, substantial heterogeneity in study populations, diet, geography, disease state, medication exposure, sample collection, sequencing methodology, bioinformatic pipelines, and taxonomic resolution limits direct comparison among studies. Fourth, microbial composition alone does not necessarily reflect functional drug-metabolizing capacity, and strain-level and temporal variation can further complicate interpretation. Finally, although this review focuses on the gut microbiome because it currently provides the strongest evidence for pharmacomicrobiomic interactions, it does not comprehensively address microbiomes at other anatomical sites or every therapeutic class potentially affected by microbial activity. These limitations highlight the need for standardized, longitudinal, functionally oriented, and clinically validated studies before many microbiome-associated drug-response signatures can be incorporated into routine precision medicine.

## 7. Conclusions

The gut microbiome represents a dynamic component of human pharmacology capable of modifying the efficacy and safety of both synthetic drugs and natural products. Through direct enzymatic biotransformation and indirect effects on intestinal barrier function, microbial metabolites, bile acid signaling, host immunity, inflammation, and hepatic drug metabolism, intestinal microorganisms can contribute substantially to interindividual variation in therapeutic response. Evidence across oncology, neurological and psychiatric disorders, cardiovascular disease, metabolic disease, and natural products demonstrates that drug response cannot always be understood exclusively through host genetics and conventional pharmacokinetic principles.

However, translation of pharmacomicrobiomics into routine clinical practice remains limited by interindividual and temporal microbiome variability, methodological heterogeneity, confounding by diet, geography, disease and medication exposure, and the predominance of associative or preclinical evidence for many proposed interactions. Future progress will require standardized longitudinal studies, mechanistic validation, multicenter clinical trials, and integration of microbial functional data with host genomics and clinical variables. Such approaches may ultimately enable microbiome-informed biomarkers and interventions that improve therapeutic efficacy while reducing toxicity.

## Supplementary Material

Supplementary Material

**Supplementary Materials:** The following supporting information can be downloaded at: https://www.mdpi.com/article/10.3390/applmicrobiol6100113/s1, Figure S1: Annual number of Google Scholar publications for the search terms “Microbiome Drug Metabolism”, “Microbiome Drug Interactions”, “Microbiome Drug Efficacy”, and “Microbiome Drug Pharmacokinetics” from 2010 to 2026. The increasing number of publications highlights the rapid growth of pharmacomicrobiomics research over the past 17 years. Searches were performed on 31 July 2026 using Google Scholar (https://scholar.google.com/).

## Figures and Tables

**Figure 1.**
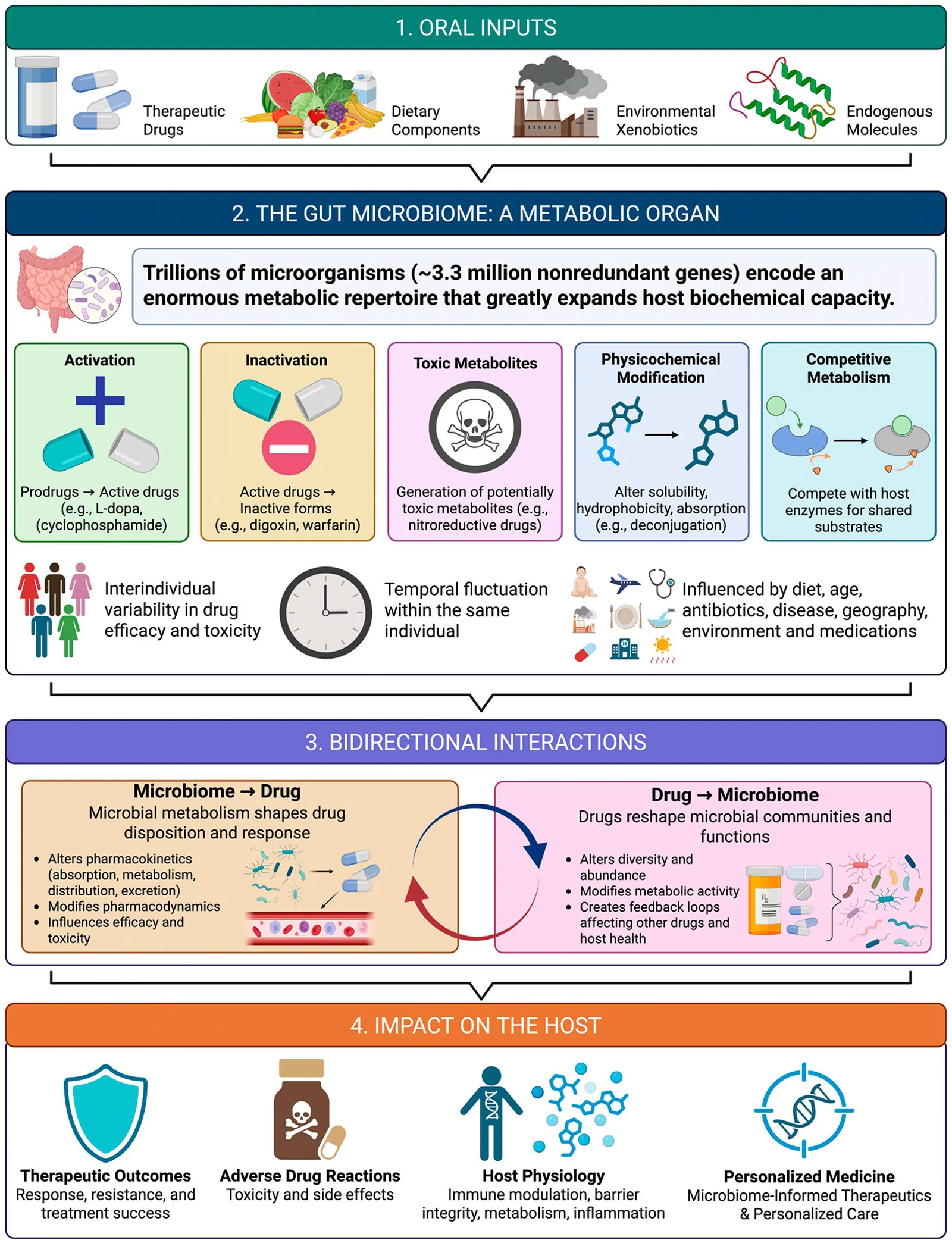
Schematic overview of the gastrointestinal microbiome as a metabolically active organ that extends the biochemical capabilities of the host. Orally administered therapeutic drugs, dietary components, environmental xenobiotics, and endogenous molecules first encounter the gut microbiome, whose extensive enzymatic repertoire mediates diverse biochemical transformations, including drug activation, inactivation, generation of toxic metabolites, physicochemical modification, and competition with host metabolic pathways. These microbial processes contribute to substantial interindividual and temporal variability in drug response and are influenced by factors such as diet, age, antibiotic exposure, disease, geography, environmental conditions, and medication history. The figure also illustrates the bidirectional nature of microbiome–drug interactions, whereby microorganisms modify drug pharmacokinetics, pharmacodynamics, efficacy, and toxicity, while drugs simultaneously alter microbial composition, diversity, and metabolic activity. Collectively, these interactions influence therapeutic outcomes, adverse drug reactions, host physiology, and the development of microbiome-informed precision medicine strategies.

**Figure 2.**
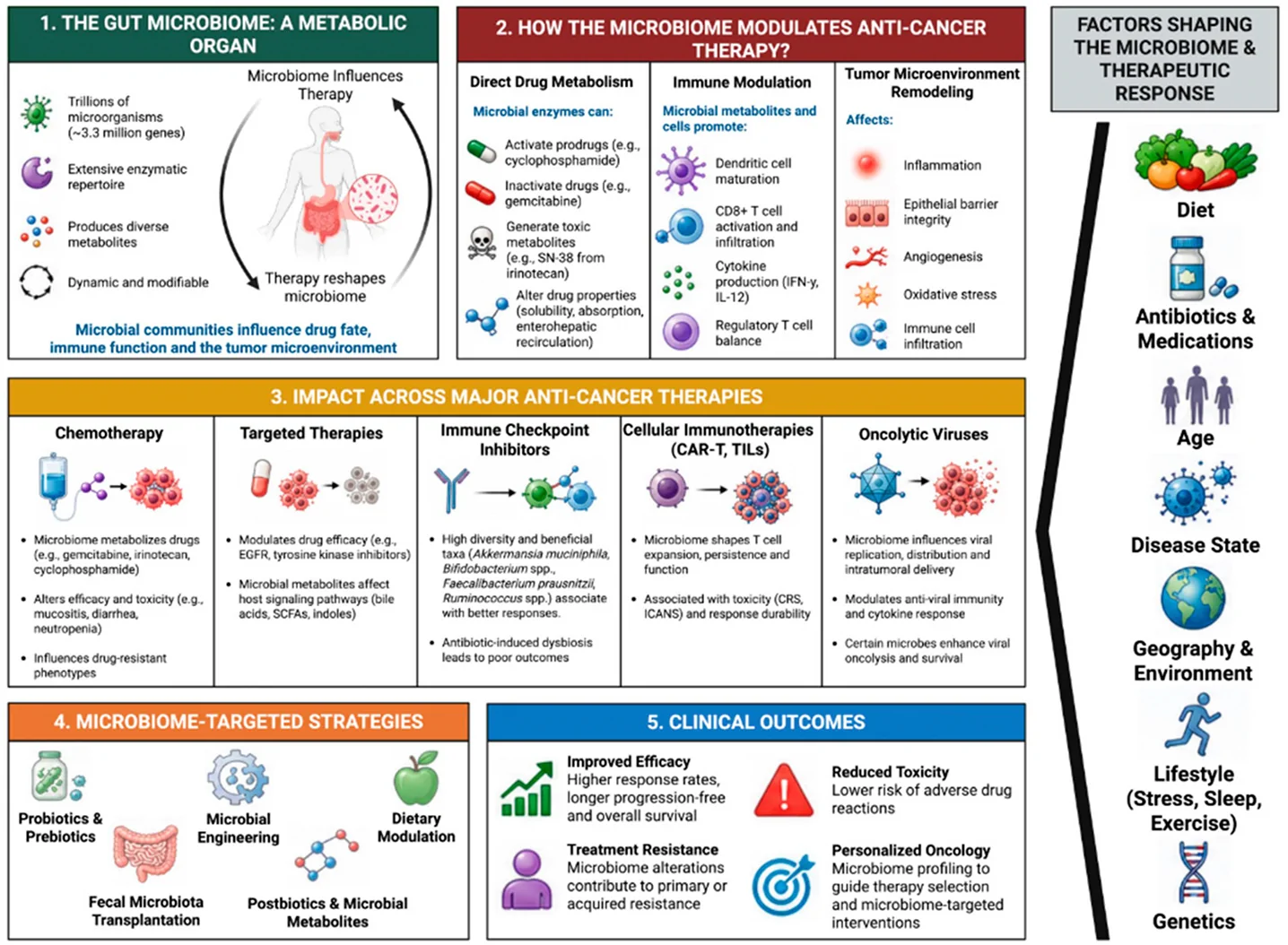
The gut microbiome is a determinant of anti-cancer drug efficacy. Overview of the multifaceted role of the gut microbiome in regulating the efficacy and safety of anticancer therapies. The gastrointestinal microbiome functions as a metabolically active organ composed of trillions of microorganisms with an extensive enzymatic repertoire capable of producing diverse metabolites that influence drug disposition, immune function, and the tumor microenvironment. The figure summarizes three principal mechanisms through which the microbiome modulates anticancer therapies: (i) direct drug metabolism, where microbial enzymes activate prodrugs, inactivate therapeutic agents, generate toxic metabolites, and alter drug physicochemical properties; (ii) immune modulation, through regulation of dendritic cell maturation, T-cell activation, cytokine production, and immune homeostasis; and (iii) tumor microenvironment remodeling, including effects on inflammation, epithelial barrier integrity, angiogenesis, oxidative stress, and immune cell infiltration. These mechanisms influence the therapeutic response to multiple classes of anticancer treatments, including conventional chemotherapy, targeted therapies, immune checkpoint inhibitors, cellular immunotherapies (CAR-T cells and tumor-infiltrating lymphocytes), and oncolytic viruses. The diagram also highlights major host and environmental factors that shape microbiota composition and function, including diet, medications, antibiotic exposure, age, disease status, genetics, lifestyle, and geographic location. Finally, the figure illustrates emerging microbiome-targeted therapeutic strategies, including probiotics, prebiotics, dietary modulation, postbiotics, microbial engineering, and fecal microbiota transplantation, that aim to improve treatment efficacy, reduce toxicity, overcome therapeutic resistance, and support microbiome-informed precision oncology.

**Figure 3.**
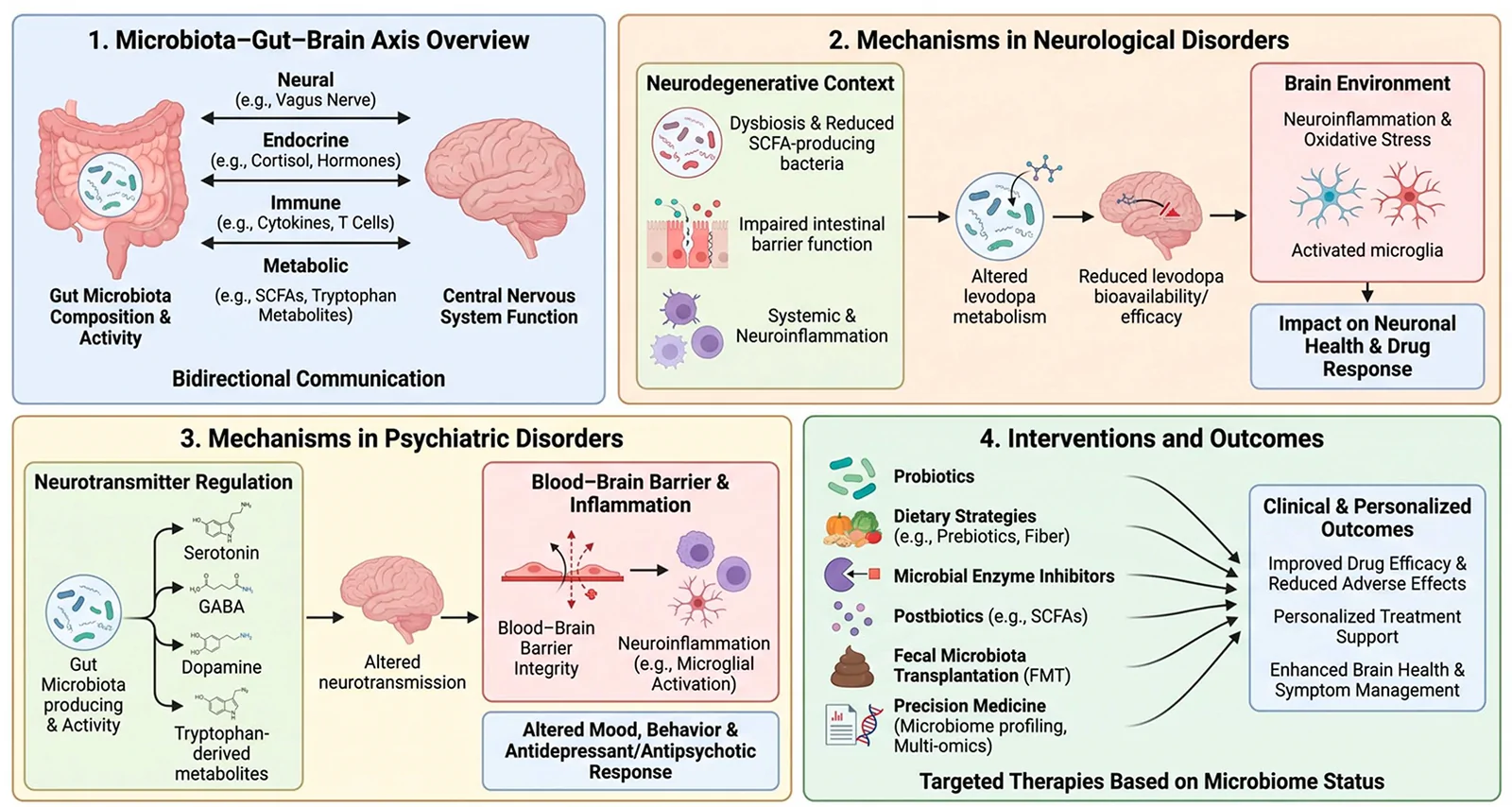
Impact of the gut microbiome on neurological and psychiatric disorders through the microbiota–gut–brain axis. The gut microbiome influences neurological and psychiatric diseases through bidirectional communication between the gastrointestinal tract and the central nervous system via neural, endocrine, immune, and metabolic pathways. In neurological disorders, microbial metabolism alters the bioavailability and efficacy of drugs such as levodopa, while dysbiosis, reduced short-chain fatty acid (SCFA)-producing bacteria, impaired intestinal barrier function, and neuroinflammation contribute to disease progression and variability in therapeutic response. In psychiatric disorders, intestinal microorganisms regulate the production of neuroactive metabolites, including serotonin, γ-aminobutyric acid (GABA), dopamine, and tryptophan-derived metabolites, thereby influencing neurotransmission, blood–brain barrier integrity, neuroinflammation, and the response to antidepressant and antipsychotic therapies. Emerging microbiome-targeted interventions, including probiotics, dietary strategies, microbial enzyme inhibitors, postbiotics, fecal microbiota transplantation (FMT), and precision medicine approaches based on microbiota profiling and multi-omics, offer opportunities to improve drug efficacy, reduce adverse effects, and support personalized treatment strategies for neurological and psychiatric disorders.

**Figure 4.**
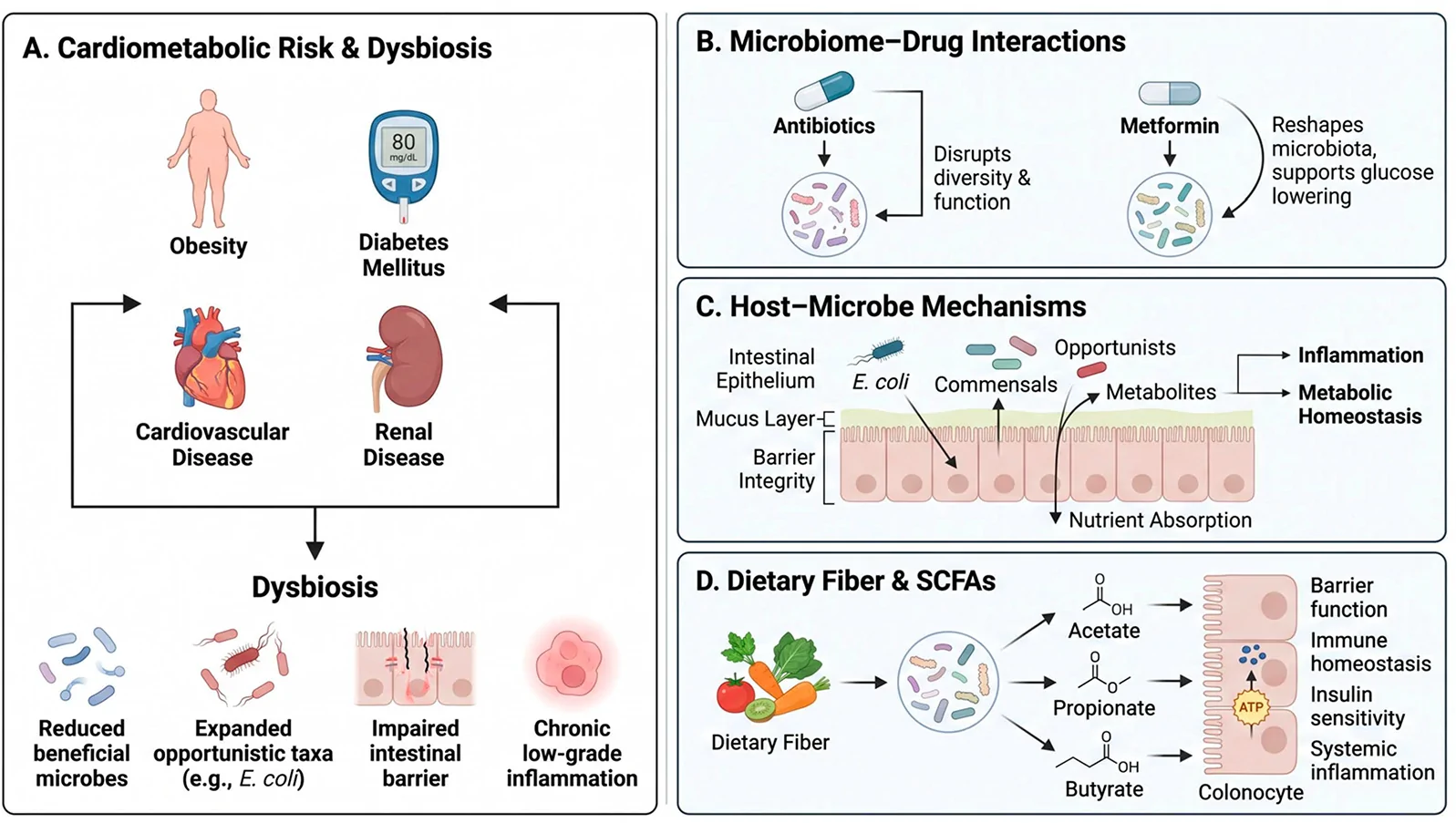
Interactions between the gut microbiome, metabolic diseases, and microbiome-mediated therapeutic responses in cardiovascular and metabolic disorders. (**A**) Major cardiometabolic risk factors, including diabetes mellitus, obesity, renal disease, and cardiovascular disease, are associated with alterations in gut microbial diversity and composition, promoting intestinal dysbiosis characterized by reduced beneficial microorganisms, expansion of opportunistic taxa, impaired intestinal barrier integrity, and chronic low-grade inflammation. These alterations contribute to disease progression while simultaneously influencing host metabolism and therapeutic responsiveness. (**B**) Bidirectional interactions between pharmacological interventions and the gut microbiome. Antibiotic exposure disrupts microbial diversity and metabolic function, whereas metformin reshapes the intestinal microbiota by increasing beneficial taxa and short-chain fatty acid (SCFA)-producing bacteria, thereby contributing to its glucose-lowering activity beyond its direct effects on hepatic gluconeogenesis. (**C**) Representative mechanisms through which intestinal microorganisms influence host metabolism. Changes in microbial composition, including the abundance of *Escherichia coli* and other commensal or opportunistic bacteria, alter epithelial barrier integrity, microbial metabolite production, and host–microbe interactions that regulate inflammation, nutrient absorption, and metabolic homeostasis. (**D**) Dietary modulation of the gut microbiome. Dietary fiber promotes microbial fermentation and SCFA production, including acetate, propionate, and butyrate, which serve as energy substrates for colonocytes, strengthen epithelial tight junctions, improve intestinal barrier function, regulate immune homeostasis, enhance insulin sensitivity, and reduce systemic inflammation.

**Figure 5.**
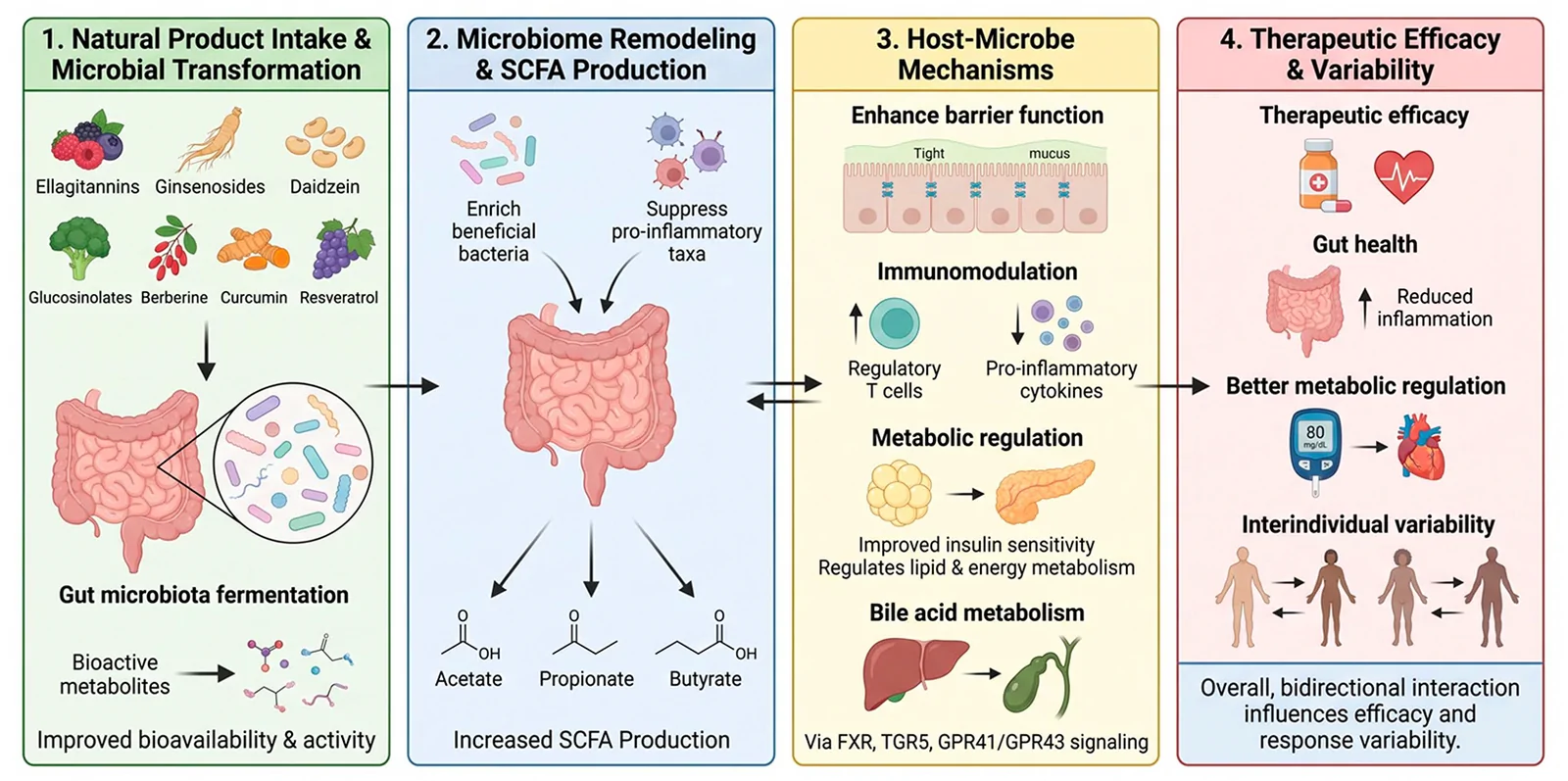
Bidirectional interactions between natural products and the gut microbiome. The gut microbiome metabolizes numerous phytochemicals, including ellagitannins, ginsenosides, daidzein, glucosinolates, berberine, curcumin, and resveratrol, into bioactive metabolites with enhanced bioavailability and biological activity. Conversely, these natural products remodel the intestinal microbiota by enriching beneficial bacteria, suppressing pro-inflammatory taxa, increasing short-chain fatty acid (SCFA) production, strengthening intestinal barrier integrity, modulating immune responses, and regulating bile acid metabolism through signaling pathways such as FXR, TGR5, and GPR41/GPR43. Together, these reciprocal interactions contribute to therapeutic efficacy and help explain interindividual variability in responses to botanical compounds.

## Data Availability

No new data were created or analyzed in this study. Data sharing is not applicable to this article.

## Abbreviations

The following abbreviations are used in this manuscript:

ABC: ATP-binding cassette
ADME: Absorption, distribution, metabolism, and excretion
AhR: Aryl hydrocarbon receptor
AI: Artificial intelligence
AKT: Protein kinase B
CAR: Constitutive androstane receptor
CAR-T: Chimeric antigen receptor T-cell
CCR9: C-C chemokine receptor type 9
CD4: Cluster of differentiation 4
CD8: Cluster of differentiation 8
cGAS: Cyclic GMP–AMP synthase
cgr: Cardiac glycoside reductase
CRISPR: Clustered regularly interspaced short palindromic repeats
CTLA-4: Cytotoxic T-lymphocyte-associated protein 4
CXCL10: C-X-C motif chemokine ligand 10
CXCR3: C-X-C motif chemokine receptor 3
CYP: Cytochrome P450
CYP1A2: Cytochrome P450 family 1 subfamily A member 2
CYP2C9: Cytochrome P450 family 2 subfamily C member 9
CYP2D6: Cytochrome P450 family 2 subfamily D member 6
CYP3A4: Cytochrome P450 family 3 subfamily A member 4
DAMPs: Danger-associated molecular patterns
FMT: Fecal microbiota transplantation
FXR: Farnesoid X receptor
GABA: γ-Aminobutyric acid
GLP-1: Glucagon-like peptide-1
GPR41: G protein-coupled receptor 41
GPR43: G protein-coupled receptor 43
GPR109A: G protein-coupled receptor 109A
HSV-1: Herpes simplex virus type 1
ICIs: Immune checkpoint inhibitors
IgA: Immunoglobulin A
IL-1β: Interleukin-1 beta
IL-6: Interleukin-6
IL-10: Interleukin-10
IL-12: Interleukin-12
IL-17: Interleukin-17
JAK: Janus kinase
L-DOPA: Levodopa (L-3,4-dihydroxyphenylalanine)
LPS: Lipopolysaccharide
MAPK: Mitogen-activated protein kinase
MHC: Major histocompatibility complex
NF-κB: Nuclear factor kappa B
OVs: Oncolytic viruses
PBPK: Physiologically based pharmacokinetic
PD-1: Programmed cell death protein 1
PD-L1: Programmed death ligand 1
PI3K: Phosphoinositide 3-kinase
PXR: Pregnane X receptor
SCFAs: Short-chain fatty acids
SLC: Solute carrier
SSRI: Selective serotonin reuptake inhibitor
STAT3: Signal transducer and activator of transcription 3
STING: Stimulator of interferon genes
TGR5: Takeda G protein-coupled receptor 5
Th1: T helper type 1
Th17: T helper 17
TMA: Trimethylamine
TMAO: Trimethylamine N-oxide
TNF-α: Tumor necrosis factor alpha
T-VEC: Talimogene laherparepvec
UAGM: Universidad Ana G. Méndez
VSV: Vesicular stomatitis virus
