# Supplementary material for: Role of the Gut Microbiome in Natural and Synthetic Drug Efficacy and Safety: A Narrative Review
Source: Appl Microbiol (Basel). Author manuscript; Available in PMC 2026 Oct 10. (PMC13652313; doi:10.3390/applmicrobiol6100113)
Supplement: Supplementary file 1 [file NIHMS2213510-supplement-Supplementary_Material.zip › applmicrobiol-4543684-supplementary.pdf]

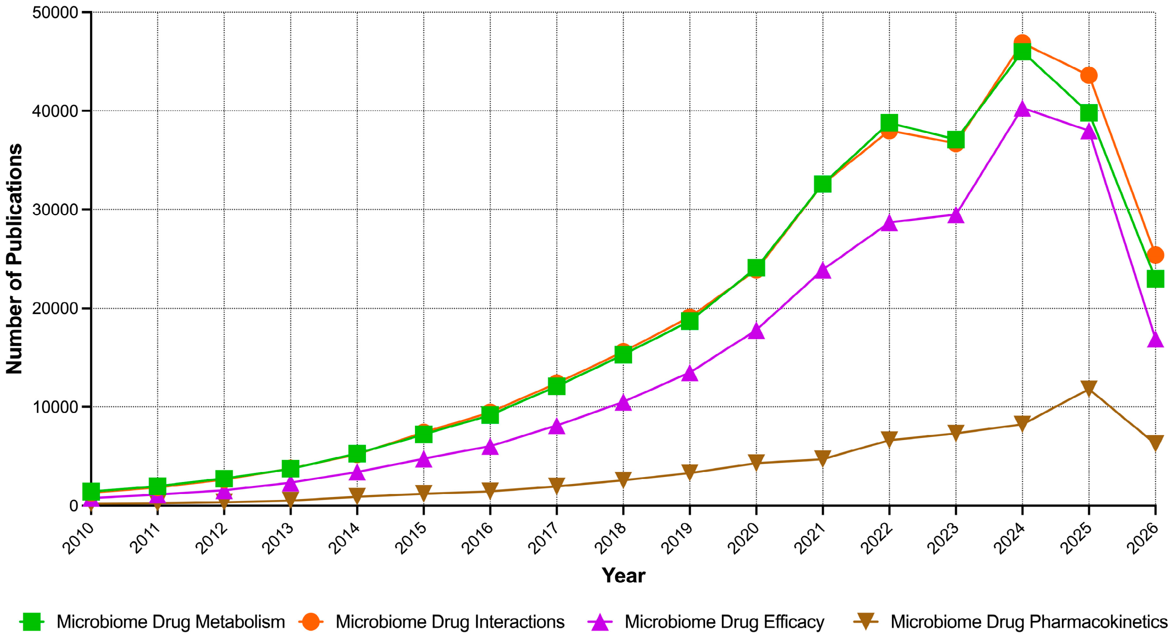

Figure S1. Annual number of Google Scholar publications for the search terms "Microbiome Drug Metabolism", "Microbiome Drug Interactions", "Microbiome Drug Efficacy", and "Microbiome Drug Pharmacokinetics" from 2010 to 2026. The increasing number of publications highlights the rapid growth of pharmacomicrobiomics research over the past 17 years. Searches were performed on 31 July 2026 using Google Scholar (https://scholar.google.com/).
